# Conceptualizing Pathways to Depression and Anxiety in Autistic Youth Using the Cognitive and Behavioral Model of Low Self-Esteem

**DOI:** 10.1016/j.cbpra.2024.11.002

**Published:** 2024-12-09

**Authors:** Matthew J. Hollocks, Jessica M. Schwartzman

**Affiliations:** Institute of Psychiatry, Psychology & Neuroscience, King’s College London, and Service for Complex Autism & Associated Neurodevelopmental Disorders, South London and Maudsley NHS Foundation Trust; Keck School of Medicine, University of Southern California, Los Angeles, and Children’s Hospital Los Angeles

**Keywords:** autism, anxiety, depression, treatment, self-esteem

## Abstract

Autistic youth are more likely to experience both anxiety and depression than their nonautistic peers, yet treatment options are extremely limited. Clinicians working with this population lack a robust evidence base of psychological models within which to formulate and treat these enduring internalizing disorders in autistic youth. Negative self-esteem is a robust risk factor—and treatment target—for internalizing disorders in nonautistic youth that remains largely understudied in autistic youth. The Cognitive and Behavioral Model of Low Self-Esteem (Fennell, 1997) was conceived to guide the development of cognitive-behavioral interventions targeting this construct in the general population. The model highlights how low-self-esteem develops from the interacting effects of temperament and negative experiences, leading to the formation of dysfunctional assumptions, and the subsequent development and maintenance of anxiety and depression. Autistic individuals are known to have specific vulnerabilities across the core elements of this model, making it potentially pertinent for this population. In this paper, we describe a theoretical extension of the model for autistic youth. We then use a blended case example to inform case conceptualization using the model to understand how low self-esteem may develop in an autistic youth and act to maintain anxiety and depression. Future directions for research into the use of the Low Self-Esteem Model in autistic youth are also discussed.

Autistic youth are at greater risk of experiencing mental health conditions than the general population (Simonoff et al., 2008). Anxiety (40–80% of autistic people experience at least one DSM-5 anxiety disorder; Hollocks et al., 2023) and depression (10% in autistic adolescents and 23% in adulthood; Hollocks et al., 2019, 2023) are particularly common in this population. With increasing prevalence rates of internalizing disorders in adolescence, there is a clear need for effective interventions for this age group and an exploration of which psychological processes may be particularly impactful on mental health. Autistic youth frequently experience anxiety and depression concurrently, which poses a clinical challenge in terms of treatment prioritization. Interfering effects of depressive cognitions on the effectiveness of anxiety interventions, and vice versa, also complicate treatment efforts. Further, there is need for a cognitive and behavioral therapy model that can account both for the common dual presentation of anxiety and depression in autistic youth and the transdiagnostic and autism-unique experiences that precipitate and/or act to maintain these internalizing symptoms. One potential psychological model, the Cognitive and Behavioral Model of Low Self-Esteem (Fennell, 1997), could provide a useful framework for guiding both clinical work and future research with autistic youth.

## Cognitive and Behavioral Model of Low Self-Esteem

The Cognitive and Behavioral Model of Low Self-Esteem was originally proposed by Fennell (1997) to establish a cognitive model for low self-esteem and to guide the development of cognitive-behavioral interventions targeting this construct. The Cognitive and Behavioral Model of Low Self-Esteem is often embedded within a broader cognitive and behavioral therapy (Rimes et al., 2023) framework to target self-esteem (i.e., mechanism) to improve internalizing symptom severity. Broadly in this model, low self-esteem (i.e., negative, persistent image of the self that shapes how one perceives and makes sense of life experiences) develops out of an interaction between temperament and negative experiences (e.g., neglect, loss, rejection, etc.). An individual then develops pessimistic beliefs about the self (e.g., “I am unlovable” or “I’m not good enough”) from repeated negative early experiences. Pessimistic beliefs then distort how the individual views themselves, others, and the world. Over time, these dysfunctional assumptions build and create unrealistic standards for that individual. For example, an individual with a negative belief that they are “unlovable” may then assume that if they do everything perfectly (e.g., at work, in relationships), then they will become worthy of love. Dysfunctional assumptions set the individual up for failure in their attempt to achieve their unrealistic standards, which then reinforces their pessimistic belief.

Given the connection between thoughts, emotions, and behaviors, the resulting emotional and behavioral consequences of these pessimistic beliefs depends on *how* the person assumes a situation will unfold. For example, the individual may encounter a situation (e.g., party) that they *anticipate* could violate their assumptions (e.g., “I’m unlovable, so I have to hide my true self at all times at this party”). The *anticipation* of a negative outcome then initiates a negative spiral of anxious thoughts, physiological responses, and behaviors. In other words, anticipating a negative outcome leads to anxiety. In contrast, the individual may encounter the same situation and *believes* that their assumptions will be violated (e.g., “I’m unlovable, so nobody will like me at this party”). The *expectation* of a negative outcome then initiates a negative spiral of *depressive* thoughts, physiological responses, and behaviors. Repeated, negative assumptions and expectations may become reinforced over time and result in more severe anxiety and/or depression.

A meta-analytic review of CBT interventions that target self-esteem using the Cognitive and Behavioral Model of Low Self-Esteem reported a wide range of intervention delivery formats, including weekly group, weekly individual, or 1-day workshop sessions (Kolubinski et al., 2018). Across studies, significant improvements in self-esteem, and reductions in anxiety and depressive symptom severities, were reported. Findings highlight the flexibility of targeting negative self-esteem using the Cognitive and Behavioral Model of Low Self-Esteem in various CBT delivery formats and the efficacy of this model in improving internalizing symptom severities. CBT-based approaches targeting self-esteem have begun to be evaluated with autistic youth and demonstrate initial promise in treating depression and anxiety in this population (Schwartzman et al., 2023; Spain & Blainey, 2017). Similarly, CBT-based approaches targeting self-esteem appear efficacious with other clinical groups (e.g., ADHD, chronic health conditions), including group-based CBT interventions targeting self-esteem (Bramham et al., 2009; Cherkasova et al., 2020). However, the application and testing of specific CBT models to autistic youth remains underresearched and is a critical extension of CBT treatment research as autistic youth are more likely to experience internalizing disorders than the general population and benefit more from adapted CBT approaches than standard ones (Sharma et al., 2021).

## Theoretical Extension of the Cognitive and Behavioral Model of Low Self-Esteem to Autistic Youth

**Fig. 1.**
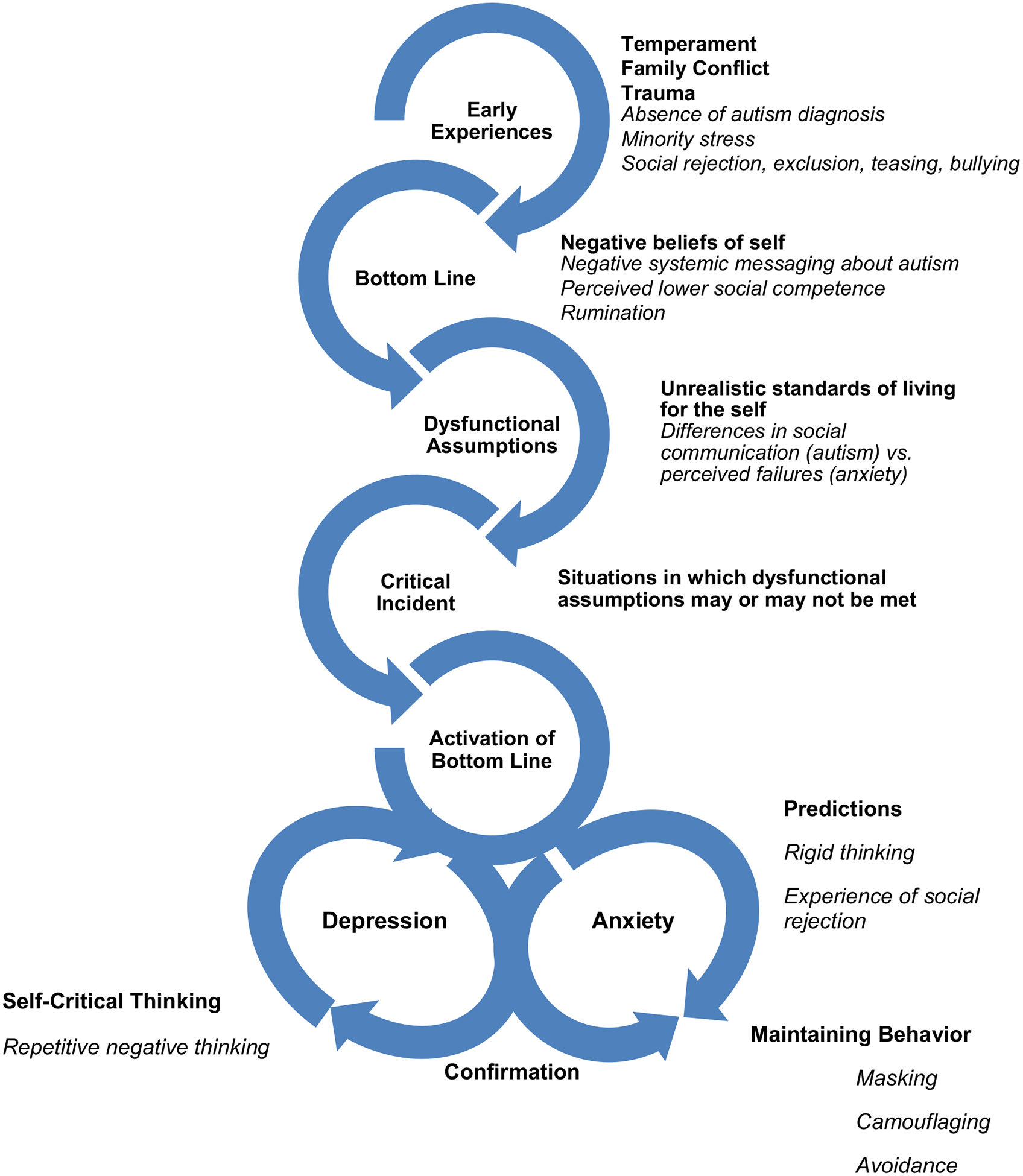
Theoretical Extension of the Cognitive and Behavioral Model of Low Self-Esteem for Use with Autistic People. Note: The original components of the Self-Esteem Model (Fennell 1997) are shown in bold, with autism specific considerations added in italics.

The low self-esteem model described above likely resonates with the experiences of autistic youth, with research indicating that they report lower self-esteem than their nonautistic peers (Van der Cruijsen & Boyer, 2021). Here, we present a theoretical version of the original Cognitive and Behavioral Model of Low-Self Esteem extended for use with autistic youth, with a visual representation in Fig. 1. It is important to state that the aim here is not to propose a comprehensive model to explain low self-esteem in autistic youth, but rather, to suggest a model for cognitive and behavioral intervention with autistic youth that can be helpful to explain the impact of unique autistic experiences on mental health trajectories in this population.

A positive identity or self-concept is important for developing and maintaining strong self-esteem (Ryeng et al., 2013) and is protective against the development of anxiety and depression (Sowislo & Orth, 2013). For autistic people, research indicates that more dissatisfaction with one’s autistic identity is related to both lower self-esteem (Corden et al., 2021) and greater anxiety and depression (Cooper et al., 2017). Relatedly, research suggests that increased masking behaviors, which are often conceptualized as a suppression of autistic traits in social settings, can also serve as additional forms of safety behaviors that reinforce anxiety (Lei et al., 2022) and depression (Hull et al., 2021). Conversely, increased awareness of, and appreciation for, one’s autistic identity may be a salient treatment target for youth with depression (Schwartzman et al., 2023) and may be vital to understanding and formulating dysfunctional assumptions. A potential strength of extending the low self-esteem model to autistic youth is the ability to integrate their early life experiences, which are likely to be implicated in the development of a positive self-concept and thus play a vital role in understanding anxiety and depression for this population. By extending this model, we are also able to integrate the role of individual differences in characteristics that are more common among autistic people (e.g., difficulties with emotional recognition and regulation, perseverative thoughts) that interact with negative experiences to impact self-esteem.

While negative experiences may be broad, we focus on several domains that occur more frequently for autistic youth and are important to extending the Cognitive and Behavioral Model of Low Self-Esteem to this population. For some autistic youth, negative experiences occur in the absence of an autism diagnosis (i.e., this is yet to be detected), providing no frame of reference for their experience (e.g., “Something about me is different, why?”). Similarly, some autistic children have negative experiences related to autism minority stress at school, home, or out in the community, with downstream effects on how they view themselves, others, and the world (e.g., “A study flyer says that they are looking for an autism cure, something must be wrong with me”). Social rejection, exclusion, teasing, and bullying are unfortunately more common experiences among autistic people than the general population (Kwan et al., 2020) that may predispose autistic youth to low self-esteem (e.g., “My classmates make fun of my D&D backpack and clothing … I’m weird for liking D&D”). In addition, autistic people are over 1.5–3 times more likely to experience trauma (e.g., physical abuse, sexual assault) and maltreatment (e.g., neglect, abandonment) than their nonautistic peers (Hibbard & Desch, 2007; Sullivan & Knutson, 2000). With higher prevalence rates of trauma among autistic people, it is likely that these negative experiences impact masking behaviors (Evans et al., 2023) and self-esteem (e.g., “I’m different so I deserved that punishment”). Further, family stress and conflict are elevated in families of autistic youth with negative consequences for youth and family members alike (Ilias et al., 2018). These early experiences, combined with findings indicating that some autistic youth have difficulties in emotion regulation skills (Cai et al., 2018), may confer additional vulnerability to developing negative-self beliefs. Clinicians looking to extend the low self-esteem model to autistic youth can assess for these negative experiences in autistic youth (e.g., clinical interviews, questionnaires, etc.) and consider their causal and/or maintaining roles in the pathway to negative self-beliefs.

As outlined above in the model, these negative self-beliefs then act to distort an individual’s perceptions of themselves, peer interactions, and the world around them. Some autistic individuals perceive themselves as having lower social competence and self-worth compared to nonautistic individuals (Nguyen et al., 2020; “I never know what to say to people, I’ll never make friends”), with this effect potentially being enhanced in females (Jamison & Shuttler, 2015). Negative expectations of social interactions, often supported by real-world experiences of rejection, likely foster dysfunctional assumptions (e.g., “If I act like everyone else, I’ll be okay”) that an autistic youth may hold themselves to across situations. Over time, dysfunctional assumptions build and create unrealistic standards for that child. For example, an autistic child with a negative belief that they are “unlovable” because of their autistic identity may then assume that if they do everything perfectly (e.g., at work, in relationships), they will become like everyone else and therefore be worthy of love. Dysfunctional assumptions set the child up for failure in their attempt to achieve these perfectionistic standards, which then reinforces their negative self-belief.

In autistic youth, this pattern can be further complicated by the need to differentiate genuine social communication differences associated with autism from perceived failures associated with anxiety in social situations, both of which can reinforce negative self-beliefs, propagate dysfunctional assumptions, and create unrealistic standards that the child tries to live up to. While these processes resemble those experienced in nonautistic youth, perceptions of autistic identity, based on experiences of minority stress and negative systemic messaging about autistic identity across settings (e.g., home, schools, workplace, etc.), likely feed into negative self-beliefs. Given the transdiagnostic nature of the self-esteem model and the multiple pathways to low self-esteem for autistic people, this model can provide an integrative framework for cognitive and behavioral treatment for anxiety and depression in this population, allowing practitioners to enhance their understanding of the experiences of being an autistic person in a world not designed for them.

### Case Example

What follows is a blended case formulation to provide a working example for extending the Cognitive and Behavioral Model of Low Self-Esteem to conceptualize internalizing disorders in autistic youth. The case formulation is based on several therapy cases in which there was a common clinical presentation of social anxiety and low mood in verbally fluent autistic youths with typical intellectual abilities. Other common aspects included a history of social difficulties and bullying, as well as minimal response to standard CBT for social anxiety or depression. It is important to note that while these themes have been common among multiple young autistic people that we have worked with, each person is an individual and we do not infer that their experiences, negative self-beliefs, and subsequent dysfunctional assumptions are universal.

Beatrice (pseudonym) is a 17-year-old cisgender female with a history of long-standing social anxiety and episodic major depression. Beatrice was not diagnosed with autism until she was 15 years old, meaning that her early experiences of social difficulty and bullying were not processed through a lens of having this understanding of why she may experience difficulties in certain situations relative to her peers. Without support, Beatrice continued to have negative social experiences and failed friendships, despite her best efforts. Beatrice concluded that when she was “herself,” people did not like her. She attributed the blame for these failed friendships to her sense of “difference.” These early experiences contributed to negative-self beliefs including: “I am not normal” and “I am a bad person.” In response to these beliefs, and to manage the continued demands to “fit in” socially, several dysfunctional assumptions (i.e., rules for living) were formed: “If I don’t show my true self, then they cannot reject me” and “I have to downplay all the good things I do, then people cannot put me down.”

Once these negative self-beliefs and dysfunctional assumptions crystallized, Beatrice then experienced social interactions as triggers for recurrent negative thoughts and opportunities for her dysfunctional assumptions to be violated. Much like in a typical social anxiety presentation (Alden et al., 2008), anticipatory negative predictions are made about future social encounters, with resulting anxiety or avoidance, confirming the belief that they are “not normal” or a “bad person.” For Beatrice, this manifested as avoidance or engaging in behaviors designed to hide her true self: Beatrice, like many autistic youth, engaged in masking and safety behaviors that led to successful social interactions in the short-term that were later dismissed with negative self-beliefs (e.g., “But it wasn’t really me that made them laugh”). As outlined in the model in Fig. 1, this confirmation of Beatrice’s negative self-beliefs then contributed to her self-critical thoughts (e.g., “I should be able to do this” or “I’m not normal” or “I have let everyone down”). Over time, self-critical thoughts resulted in periods of low mood and the risk of a depressive episode for Beatrice.

Through a process of collaborative empiricism, it was possible to jointly identify several characteristics associated with autism in sessions with Beatrice, which contributed as precipitating and maintaining factors to her low self-esteem. First, social communication differences that were present in childhood, but not formally identified as autism until later adolescence, meant that social interactions were genuinely challenging for Beatrice; without a diagnosis and understanding of autism, she internalized these challenges. To manage this, Beatrice adopted masking behaviors to help her “get by” socially; at the same time, these behaviors also likely reinforced her negative self-beliefs (e.g., “I am not normal”). Cyclical negative self-beliefs likely prevented Beatrice from having the experience of genuine positive social experiences. A more rigid style of thinking (Lei et al., 2022) meant that Beatrice struggled to identify and adopt alternative perspectives and behaviors to achieve her social goals. Further, Beatrice, like some autistic youth, maintained a repetitive thinking style that can confer additional risk for rumination and periods of low mood (Gotham et al., 2014).

Without an understanding of this constellation of early traumatic experiences, autistic traits, and their resulting impact on Beatrice’s self-esteem, standard CBT approaches (i.e., exposure work; behavioral experiments targeting appraisals of social situations) were not optimal for her. For example, standard cognitive restructuring techniques may assume that Beatrice’s appraisal of a social situation (e.g., “People don’t like me, it must be because I’m different”) was incorrect and a cognitive distortion (i.e., black/white thinking). However, Beatrice, like many autistic people, experiences *actual* minority stress, social rejection, and invalidation in social situations; thus, her appraisals are accurate. Therefore, to appropriately understand Beatrice’s experiences as an autistic person (e.g., minority stress, social rejection, etc.), the clinician refrained from conceptualizing her thoughts as a cognitive distortion and instead recognized and validated her experiences. The clinician used an adapted cognitive restructuring technique to shift away from the accuracy/inaccuracy of an appraisal and towards the validation/recovery of these experiences (e.g., “Autistic people experience invalidation in social situations and your experiences are real and painful. You are persisting amidst a social world that is not designed for autistic people”). However, in some specific scenarios (e.g., worries attributed to social anxiety rather than minority stress), more traditional cognitive restructuring techniques, particularly around identifying and working with all-or-nothing thinking, are likely to be both appropriate and effective.

Therefore, the treatment with Beatrice was focused on applying this extended version of the Cognitive and Behavioral Model of Low Self-Esteem in treatment to address negative self-beliefs, dysfunctional assumptions, and negative predictions about possible violations of her rules for living that led to (and maintained) anxiety and depression. The treatment consisted of five main elements: (1) psychoeducation around autism (e.g., autistic traits, minority stress, invalidation, etc.) and links to mental health, (2) developing a shared formulation of Beatrice’s difficulties and the role of autistic traits and minority stress (both now and longitudinally), (3) increasing meta-cognitive awareness of the potential impact of autistic traits and unique experiences on thoughts, emotions, and behaviors (e.g., thought diaries, in-session discussions and reflections), (4) behavioral experiments to challenge dysfunctional assumptions and predictions, and (5) cognitive restructuring focusing on self-critical beliefs. A vital component of this work was coming to a “shared understanding” of how negative experiences had precipitated and maintained Beatrice’s difficulties. For example, the clinician supported Beatrice to identify when and how perseverative thoughts occur (i.e., increasing meta-cognitive awareness), which resulted in several insight moments and opportunities for behavioral change. Similarly, the clinician encouraged discussions around Beatrice’s social communication differences and how these manifest in social interactions, which was extremely powerful in increasing her engagement in more traditional aspects of CBT (e.g., behavioral experiments, cognitive restructuring).

Vitally, this model was combined with psychoeducation around autism and autistic experiences (e.g., masking, minority stress, etc.) to not only foster and validate her autistic identity formation, but also to identify and understand some traits that may maintain anxiety and depressive symptoms. For clinicians, a key decision point in sessions is to identify when to focus on self-exploration and validation of autism-related experiences, and when to focus on challenging dysfunctional assumptions related to anxiety and depression and focus on cognitive restructuring. To better understand the client’s lived experiences, an initial step in this decision point may be for clinicians to ask the client about their experiences of rejection, marginalization, and discrimination because of being autistic in a nonautistic world. Clinicians can validate the causal and/or maintaining role of marginalization experiences in the client’s thoughts and discuss ways to challenge assumptions, utilize cognitive restructuring, and begin to develop skills for improving self-esteem. Working within the framework of the self-esteem model focuses the attention of the therapy onto cognitive and behavioral factors that are maintaining anxiety and depression, while acknowledging that those experiences may be associated with autistic experiences and marginalization. It is at this point that working within a CBT model may differ from other frameworks that could be used to understand low self-esteem in autistic individuals. For instance, as touched upon above, the minority stress model has been used to conceptualize adverse mental health outcomes among autistic people (Botha & Frost, 2020) and remains a key model for continued investigation in this population. While the minority stress model may help formulate and understand the experiences of autistic individuals, it does not provide a clear process for treatment; however, we recognize this could be a fruitful area for expansion of this work. Critically, the framework provided by the minority stress model could guide clinicians in considering the minoritized experiences of autistic individuals and how these experiences contribute to negative self-beliefs and dysfunctional assumptions.

### Future Research

The theoretical extension of the Cognitive and Behavioral Model of Low Self-Esteem (Fennell, 1997) to autistic youth presented here, accompanied by a case example, lays an important foundation for continued research in this domain. Recommendations for future research on the model in autistic people include a systematic investigation of each component of the model, in collaboration with autistic people, to elucidate potential pathways to well-being and/or anxiety and depression. Co-creation with autistic people is necessary to examining each component to not only identify shared experiences with nonautistic people (e.g., early experiences of bereavement, lack of praise), but also to update each component with autism-specific experiences (e.g., minority stress, social camouflaging, autistic burnout, etc.). Once designed, the updated model could be tested cross-sectionally and longitudinally with autistic youth and adults to understand the emergence and maintenance of low self-esteem and associations with anxiety and depression. These mechanistic findings could then be leveraged to enhance case conceptualizations and intervention approaches for depression and anxiety in autistic people, affording a more tailored approach to mental healthcare.
